# Tau secretion is correlated to an increase of Golgi dynamics

**DOI:** 10.1371/journal.pone.0178288

**Published:** 2017-05-26

**Authors:** Nguyen-Vi Mohamed, Alexandre Desjardins, Nicole Leclerc

**Affiliations:** Département de Neurosciences, Université de Montréal, Centre de Recherche du Centre Hospitalier de l’Université de Montréal Hospital (CRCHUM), Montréal, Québec, Canada; National Center for Geriatrics and Gerontology, JAPAN

## Abstract

Tau protein can be released by neurons, an event linked to the propagation of Tau pathology in Alzheimer’disease (AD). Neuronal hyperexcitability was shown to significantly increase Tau release by neurons. We confirmed this in the present study. In a previous study, it was demonstrated that hyperexcitability induces Golgi apparatus dynamics resulting in its fragmentation. Our present results revealed that the increase of Tau secretion upon hyperexcitability could be significantly reduced by preventing Golgi dynamics through the inactivation of cdk5. We then verified whether a Golgi fragmentation not induced by hyperexcitability could also increase Tau secretion. The suppression of Rab1A, Rab GTPase associated with the Golgi membranes, known to induce a Golgi fragmentation increased Tau secretion by both neurons and HeLa cells. Although it remains to be demonstrated whether the Golgi is directly involved in Tau secretion, the present results demonstrate that its dynamics are correlated to a modulation of Tau secretion.

## Introduction

Tau protein that becomes misfolded and aggregated forms the neurofibrillary tangles (NFTs) that propagate in a predictable manner in AD brain [1–4]. The mechanisms leading to the spreading of NFTs in human brain remain elusive. A new concept has emerged that in several neurodegenerative diseases including AD, the spreading of misfolded protein aggregates would occur by cell-to-cell transmission [5–7]. In such a case, NFTs would propagate in the brain by the release of misfolded TAU aggregates from an affected neuron followed by its uptake in neighboring neurons. Consistent with this, several studies have reported that Tau can be secreted and endocytosed by neurons both in vitro and in vivo [8–21]. In a transgenic mouse model where human TAU overexpression was restricted to the entorhinal cortex, the spreading of Tau pathology was observed along synaptically connected circuits [22, 23]. More recently, it was reported that synaptic contacts favor cell-to-cell propagation of Tau pathology implying that secretion of Tau by pre-synaptic neurons and its uptake by post-synaptic neurons would underlie the propagation of Tau pathology in the brain [24].

The presence of Tau in the interstitial fluid in Tau transgenic mice brain and in the cerebrospinal fluid (CSF) of Tau transgenic mice before neurodegeneration indicate that extracellular Tau can be released by an active process of secretion in vivo [11, 25]. As noted for other proteins involved in neurodegenerative diseases, Tau seems to be released by unconventional secretory pathways [9, 12, 16, 26–28]. However, the mechanisms regulating Tau secretion remain to be elucidated. In recent studies, Tau was shown to be secreted by primary cortical neurons upon neuronal activity both in vitro and in vivo [29, 30]. In particular, neuronal hyperexcitability observed at early stages of both sporadic and familial AD and in AD mouse models was shown to increase Tau release [30–35].

A recent study reported that hyperexcitability induces a fragmentation of the Golgi [36]. The Golgi, involved in both conventional and unconventional secretion, is located near to the nucleus where it is organized in stacks of flattened cisternae [37, 38]. The Golgi is highly dynamic and can be fragmented under both physiological and pathological conditions [39, 40]. Most interestingly, a fragmentation of the Golgi was noted at early stages of several neurodegenerative diseases [41–44]. In AD, this fragmentation was correlated to the accumulation of hyperphosphorylated TAU [45]. We reported a similar observation in Tau transgenic mice [46]. In the present study, we investigated whether the Golgi fragmentation induced by hyperexcitability had an impact on Tau secretion in primary cortical neurons. Our data revealed that the release of Tau was decreased when the Golgi dynamics were blocked. In both neurons and HeLa cells overexpressing human TAU, the induction of Golgi fragmentation increased Tau secretion. Collectively, our data demonstrate that Golgi dynamics are correlated to a modulation of Tau secretion. In AD, the Golgi fragmentation observed at early stages of disease could correspond to the increase of TAU in the CSF of patients.

## Materials and methods

### Cell culture

The use of animals and all surgical procedures described in this article were carried out according to *The guide to the Care and Use of Experimental Animals of the Canadian Council on Animal Care*. The ethical approval was obtained from the Animal care and ethics committee of the Centre de Recherche du Centre Hospitalier de l’Université de Montréal (CRCHUM) (protocol number N14002 NLs). Primary cortical cultures were prepared from E18 Sprague Dawley rat embryos (Charles River, Montréal, Québec, Canada). To collect the embryos, the rat was euthanized in a CO_2_ chamber. The cerebral cortices were treated with trypsin (0.025% at 37°C for 20 min). The reaction was stopped with trypsin inhibitor solution containing DNAse. Neurons were dissociated by several passages through a Pasteur pipette. The cells were then plated either on glass coverslips or on culture dishes coated with poly-D-lysine (Sigma, Oakville, ON, Canada). The neurons were maintained in neurobasal medium (Invitrogen, Burlington, ON, Canada) supplemented with glutamax (Invitrogen) and B27 (Invitrogen). HeLa cells (ATCC, Manassas, VA, USA) were cultured in EMEM supplemented with L-glutamine (ATCC, Manassas, VA, USA) and with 10% foetal bovine serum (Hyclone) (Thermo scientific) at 37°C in a humidified 5% CO_2_ incubator.

### Treatment with KCl, olomoucine and roscovitine

Seven days after plating, neurons were incubated in neurobasal medium containing either 10mM or 20mM of KCl for 6 hours (h) to induce hyperexcitability. Control neurons were incubated in neuronal medium containing 5mM of KCl for 6 h. Then, the medium was collected and the cells lysed. To block the fragmentation of the Golgi induced by elevated concentrations of KCl, neurons were pre-incubated either with 10μM of olomoucine (Cayman) or 2μM of roscovitine (Santa Cruz) for 1 h.

### Transfection of primary cortical neurons with *Rab1A* siRNAs and GFP-Rab1A

Four days after plating, rat cortical neurons were treated with 1μM of Accell rat Smartpool siRNA for Rab1A (E-100109-00-0010, Thermo Scientific Dharmacon). Neuronal cell lysate and medium were harvested 4 days later. The siRNA oligos were as follows:

*Rab1A* siRNA: 5’-CGGAGAAGUCCAAUGUUAA-3’

*Rab1A* siRNA: 5’-CUGUCAGUUUCCAUGCAUA-3’

*Rab1A* siRNA: 5’-GUGGUUGGUUAGAAUAUAU-3’

*Rab1A* siRNA: 5’-UUUGUGUGCUGGUUUAUAA-3’

A Accell non-targeting siRNA pool (D-001910-10-05, Thermo Scientific Dharmacon) was used as control.

Seven days after plating, neurons were transfected with GFP-Rab1A using lipofectamine 2000 (Thermo Scientific). Twenty-four h later, neurons were fixed and processed for immunofluorescence.

### Transfection of HeLa cells

Twenty-four h after plating, HeLa cells were transfected either with 100nM of *Rab1A* siRNA or 100nM of control siRNA using DermaFECT transfection reagent (Thermo Scientific Dharmacon). Twenty-four h later, the cells were transfected with wild-type TAU4R (Flag-TAU4R) using Genejuice transfection reagent (Millipore). Forty-eight h after TAU transfection, the culture medium was collected and the cells were either lysed or fixed. The medium was centrifuged at 3000 RPM for 10 min at room temperature (RT) to remove cell debris, resuspended in Laemmli buffer 1x and boiled for 5min. A human ON-TARGETplus SMARTpool siRNA was used to knockdown RAB1A in HeLa cells (L-008283-00, Thermo Scientific Dharmacon). The *RAB1A* siRNA oligos were as follows:

*RAB1A* siRNA: 5’CAGCAUGAAUCCCGAAUAU-3’

*RAB1A* siRNA: 5’GUAGAACAGUCUUUCAUGA-3’

*RAB1A* siRNA: 5’GGAAACCAGUGCUAAGAAU-3’

*RAB1A* siRNA: 5’UGAGAAGUCCAAUGUUAAA-3’.

These *RAB1A* siRNA targeted *RAB1A* mRNA at positions 392–410, 867–885, 839–857, 941–959, respectively. A non-targeting siRNA pool (D-001810-10-20), Thermo Scientific Dharmacon) was used as control.

### Preparation of cell lysates

After the culture medium was collected, the cells were immediately washed twice with PBS and once with PBS containing 0.5M NaCl to detach proteins non-specifically attached to the cell surface. The neurons were then lysed in modified RIPA buffer [Tris HCl pH 7.5 50mM, NaCl 150mM, Triton X-100 0.5%, sodium deoxycholate (SDC) 0.5%, sodium dodecyl sulfate (SDS) 1%, dithiothreitol (DTT) 1mM, NaF 50mM, Na3VO4 5mM] containing a protease inhibitor cocktail (Complete EDTA-free from Roche Diagnostics, Indianapolis, IN) and phosphatase inhibitor cocktail 2× (PhosSTOP from Roche Diagnostics). The HeLa cells were lysed in buffer containing NaCl 150mM, Tris HCl pH 7.5 50mM, Triton X-100 1%, SDS 0,1%, SDC 1%, and a protease inhibitor cocktail. Proteins were quantified using Bio-Rad DC Protein assay (Bio-Rad Laboratories Ltd., Mississauga, ON, Canada). The lysates were mixed with laemmli buffer 1x and boiled for 5 min.

### Immunoprecipitation of Tau from neuronal medium

After treatment, the culture medium of control and treated neurons was collected and centrifuged at 3000 RPM for 10 min at RT to remove cell debris. To analyze the amount of secreted Tau, Tau was immunoprecipitated from the culture medium. For each condition, 60 μl of magnetic beads coupled with anti-mouse antibodies (Invitrogen, Dynabeads^®^ M-280 Sheep anti-Mouse IgG) were washed in PBS containing 0.1% BSA and incubated overnight (O/N) at 4°C with 0.4μg of the anti-Tau antibody Tau-5 (Invitrogen #AHB0042, 1:1000). The beads were then washed and incubated for 2 h at 4°C with 1.5 ml of the culture medium. The complex bead-antibody-antigen was then washed in PBS, resuspended in 20μl of Laemmli buffer and boiled for 5 min.

### Mesoscale immunoassay to measure Tau in the neuronal cell culture

The culture medium of control and treated cells was collected and centrifuged at 3000 RPM for 10 min at RT to remove cell debris. The amount of Tau in the medium was determined by MSD MULTI-SPOT mouse total Tau assay (Meso Scale Discovery, K151DSD-1), an immunoassay platform allowing the detection of Tau in biological samples. The MSD’s electrochemiluminescence detection technology employs SULFO-TAG labels, which emit light upon electrochemical stimulation initiated at the electrode surfaces of MULTI-SPOT microplates. This sensitive assay was used according to the manufacturer's instructions. For the experiments of Tau secretion, all replicates were analyzed in a 96-well 4-spot mouse total Tau plate at the same time. The plate was read with Sector Imager 6000, a Meso Scale Discovery instrument.

### Analysis of cell membrane integrity

Cell membrane integrity was assessed by the measurement of the LDH activity in the culture medium using the LDH cytotoxicity assay kit from Cayman Chemical Company (Ann Arbor, MI, USA), according to the manufacturer's instructions. The LDH content in the samples was measured with a BIO-TEK Elx800 plate reader.

### Immunoblotting and dot blot

Equal amounts of proteins (10 μg) were loaded in each lane and electrophoresed on a 10% polyacrylamide gel with molecular weight markers (Biorad #1610374). Following SDS-PAGE separation, proteins were electrophoretically transferred to a nitrocellulose membrane. Then the membranes were incubated in 5% milk diluted in 0.2% Tween-20 Tris buffered saline (TBST) for 1 h. The nitrocellulose stripes were incubated with the primary antibodies O/N at 4°C. The antibodies used were: total Tau (DAKO #A0024, 1:15000), Tau1 (Millipore #MAB3420, 1:1000), Phospho-T181 (Invitrogen #MN1050, 1:1000), Phospho-S404 (Biosource #44-758G, 1:1000), Phospho-S199/202 (Thermo Scientific #44-768G, 1:1000), Phospho-T205 (Thermo Scientific #44-738G, 1:500), Rab1A (Santa Cruz sc-311, 1:100) and p35 C-19 (Santa Cruz #sc-820, 1:100). The membranes were then washed in TBST and incubated with the peroxidase-conjugated secondary antibodies. The membranes were again washed and revealed by chemiluminescence (Amersham Pharmacia Biotech, Quebec, Quebec, Canada). After incubation with Tau antibodies, membranes were revealed using an anti- γ-actin antibody (1–24) (Santa-Cruz #sc-65635, 1:5000) to monitor protein loading. For quantification of the immunoreactive bands, western blot image acquisition was performed using a ChemiDoc MP system (Biorad) and densitometry analysis was done with Image Lab software (version 5.1).

For staining of phospho-GM130 by dot blot, cell lysates (30 μg of proteins) prepared either from control neurons or neurons treated with 20mM KCl for 6 h were loaded on a nitrocellulose membrane placed in the dot blot apparatus. Blot was revealed with an antibody directed against GM130 phosphorylated at serine 25 (Santa Cruz #sc-377549, 1:50) diluted 1:50 in 5% BSA/PBS.

### Immunofluorescence and quantification of the Golgi area

HeLa cells and neurons were fixed in 4% paraformaldehyde prepared in PBS for 30 min. Cells were then permeabilized with 0.2% Triton X-100 in PBS for 5 min. Cultures were kept in PBS until they were processed for immunofluorescence. For immunofluorescence, coverslips were blocked with 5% normal goat serum (Invitrogen) in PBS. Then coverslips were stained with an antibody directed against total tau (DAKO #A0024, 1:500), an antibody against the Golgi marker, GM130 (BD Biosciences #610823, 1:50) and an antibody against GM130 phosphorylated at serine 25 (Santa Cruz #sc-377549, 1:50). After 3 washes in PBS, coverslips were incubated with either an anti-rabbit antibody coupled to FITC (1:500, Jackson ImmunoResearch), with an anti-rabbit antibody coupled to Alexa 350 (1:500, Invitrogen) or anti-mouse antibody coupled to Rhodamine (1:500, Jackson ImmunoResearch). The antibodies were diluted in the blocking solution. Incubations were carried out at RT for 1 h. Coverslips were then washed in PBS and mounted in mowiol. Labeled cells were visualized with an axioplant Zeiss fluorescence microscope using ×63 objective.

The surface of the Golgi was analyzed using the ImageJ software. After calibration, the tool freehand was used to delimitate the surface of the cell soma containing Golgi-positive structures detected with an antibody directed against GM130. The surface was automatically calculated by the software based on the calibration. The Golgi area was analyzed in at least 30 cells for each set of experiments and each condition. The mean of the Golgi surface area was used for comparison among the different conditions.

### Quantification of phospho-GM130 staining

The fluorescent intensity of phospho-GM130 staining was measured with the software Image J using the protocol published by the University of Chicago Integrated Light microscopy Core (https://digital.bsd.uchicago.edu/image_j.php). The color images were converted to 8-bit grayscale images. Then a duplicate of the image was used to create a binary version of the image. The threshold was adjusted to highlight the phospho-GM130 signal in neurons. To threshold all the images the same way for the multiple n, the threshold was set to type in a known range of pixel intensities. Then the particles were analyzed with the same range of calibration from one image to another and the intensity was evaluated by the mean gray value redirected to the original 8-bit grayscale image. A copy of the image was made and all counted particles were shown as numbered outlines.

### Statistical analysis

Statistical significance was evaluated with a two-tailed unpaired t-test when two conditions were compared. A one-way analysis of variance (ANOVA) test followed by a Tukey's multiple comparison test was performed for more than two groups comparison. The statistical analysis was performed using Prism 6.0c software.

## Results

### Increase of tau secretion induced by hyperexcitability is correlated to increased Golgi dynamics

**Fig 1:**
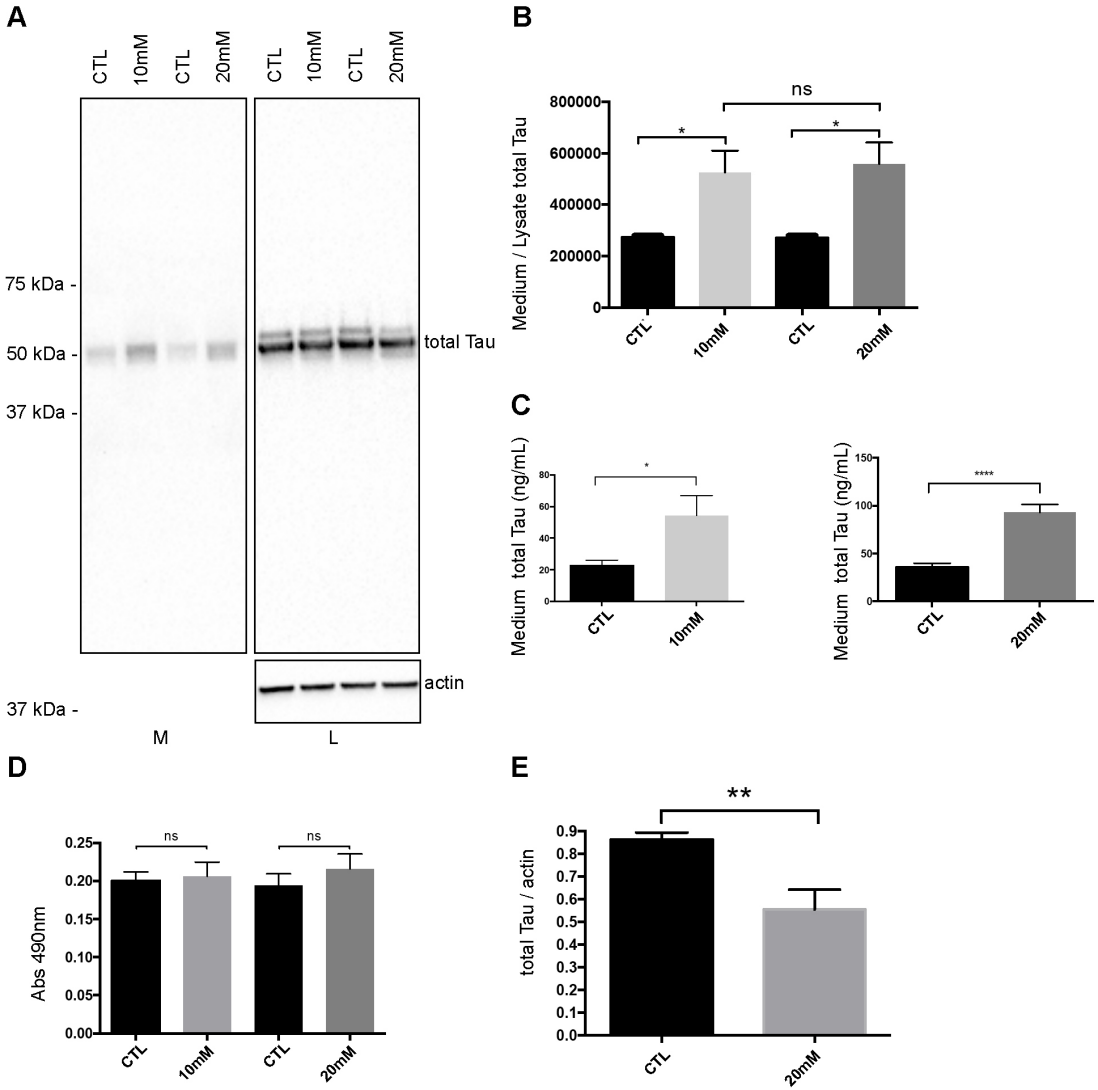
Neuronal hyperexcitability increases Tau secretion. (A) Immunoblot analysis of Tau secretion by rat neurons cultured in a medium containing either a physiological concentration of KCl (5mM, CTL) or an elevated concentration of KCl (10mM or 20mM). Tau secretion was increased by elevated concentrations of KCl. The medium (M) and cell lysate (L) were revealed with the anti-Tau antibody A0024. (B) Quantification by densitometry (arbitrary unit) of the ratio of total Tau in the medium to total Tau in the cell lysate. The amount of total Tau in the cell lysate was normalized to that of actin. (n = 7, mean ± SEM, one-way ANOVA, Tukey’s test, *P < 0.05). (C) The amount of Tau in the medium was quantified using MSD MULTI-SPOT mouse total Tau assay. (n = 5 for 10mM, n = 16 for 20mM, mean ± SEM, unpaired t-test two tailed,*P < 0.05, ****P < 0.0001) (D) Cell damage was evaluated by measuring the amount of LDH released in the culture medium. The increase of LDH in the medium was not statistically different (n.s) between control and treated neurons (n = 7, mean ± SEM, one-way ANOVA, Tukey’s test). (E) Quantification by densitometry (arbitrary unit) of total Tau in the cell lysate of control neurons and neurons treated with 20mM KCl. The amount of total Tau in the cell lysate was normalized to that of actin (n = 7, mean ± SEM, unpaired t-test two tailed, **P < 0.01).

It was previously reported that neuronal hyperexcitability increases Tau secretion [29, 30, 35]. We confirmed it in primary cortical neurons. To induce hyperexcitability, cortical neurons were treated either with 10mM or 20 mM of potassium chloride (KCl) for 6 h at 7 days after plating. Control neurons were incubated in neuronal medium containing 5mM of KCl. To evaluate the amount of secreted Tau, Tau was immunoprecipitated from the medium for its analysis by western blotting. This approach allowed us to examine the pattern of Tau in the medium (Fig 1A). As previously reported, in physiological conditions, a Tau-positive band at 52 kDa was detected in the medium by the anti-Tau antibody A0024 recognizing total Tau [10]. No oligomers and truncated forms of Tau were detected in the medium of both control and treated neurons (Fig 1A). When neurons were treated either with 10mM or 20mM KCl, this band was significantly increased compared to control neurons (Fig 1B and S1A Fig). The amount of Tau in the medium was normalized to that of total Tau in the cell lysate, which was normalized to that of actin. To quantify the amount of Tau in the medium, we used MSD MULTI-SPOT mouse total Tau assay (Fig 1C). Both approaches led us to conclude that the secretion of Tau was increased by elevated concentrations of KCl. To eliminate the possibility that the increase of Tau in the medium was caused by cell damage induced by hyperexcitability, the amount of lactate dehydrogenase (LDH), a marker of cell membrane integrity, was measured in the culture medium [47]. No significant difference was noted between control neurons and neurons incubated with elevated concentrations of KCl indicating that the increase of Tau in the medium did not result from cell damage (Fig 1D). Unexpectedly, we noted a decrease of total Tau in the cell lysate of neurons treated with 20mM KCl (Fig 1E). Collectively, the above observations confirmed that Tau secretion was significantly increased by hyperexcitability.

**Fig 2:**
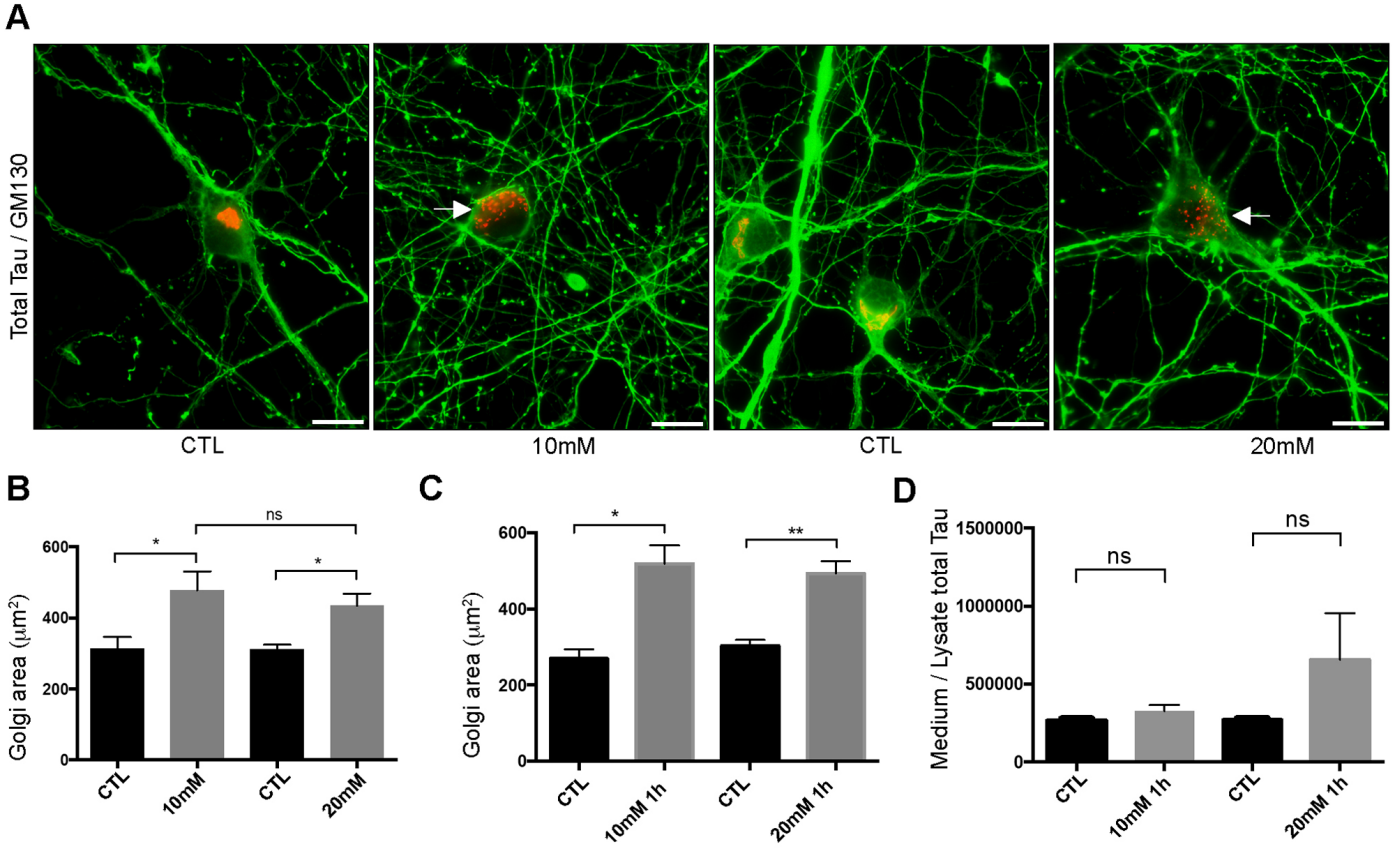
Neuronal hyperexcitability induces Golgi fragmentation. (A) Immunostaining of the GM130 (red) antibody and the A0024 antibody (green) directed against total Tau. The Golgi apparatus was fragmented when neurons were incubated with elevated concentrations of KCl concentrations (arrows). Scale bar = 30μm. (B) Quantitative analysis of the surface area of the Golgi complex (μm^2^) either upon a physiological concentration of KCl or high concentrations of KCl revealed that hyperexcitability increased Golgi fragmentation. At least 30 cells were evaluated for each condition and in each set of experiments (n = 6, mean ± SEM, one-way ANOVA, Tukey’s test, *P < 0.05). (C) Quantitative analysis of the surface area of the Golgi in neurons treated either with a physiological concentration of KCl or a high concentration of KCl for 1h revealed an increase of Golgi fragmentation. At least 30 cells were evaluated for each condition and in each set of experiments (n = 6, mean ± SEM, one-way ANOVA, Tukey’s test, *P < 0.05, **P<0.01). (D) Quantification by densitometry (arbitrary unit) of the ratio of total Tau in the medium to total Tau in the cell lysate of neurons treated either with a physiological concentration of KCl or a high concentration of KCl for 1h. The amount of total Tau in the cell lysate was normalized to that of actin. (n = 6, mean ± SEM, one-way ANOVA, Tukey’s test)

Hyperexcitability was reported to induce a fragmentation of the Golgi [36]. We investigated whether the increase of Tau secretion was linked to this event. Before addressing this point, we confirmed that the Golgi was fragmented upon hyperexcitability as previously reported. Neurons incubated with elevated concentrations of KCl were fixed and processed for immunofluorescence using an antibody directed against GM130, a marker of the cis-Golgi [48]. In control neurons, the Golgi appeared as a compacted tubular structure near the nucleus whereas in treated neurons, the Golgi was fragmented in multiple disconnected dots dispersed in the cell body (Fig 2A). Consistent with this reorganization, the Golgi area was significantly increased when neurons were treated either with 10mM or 20 mM KCl for 6 hours (Fig 2B and S1B Fig). The percentage of neurons presenting a fragmented Golgi was increased upon treatment of KCl. Only 7% of control neurons had a fragmented Golgi compared to 41.2% and 50.2% of neurons treated with 10mM and 20mM of KCl, respectively. We then examined whether Golgi fragmentation occurred either before or in parallel to tau secretion. Interestingly, the fragmentation was observed as early as 1 h after KCl (10 and 20 mM) was added (Fig 2C). At this time point, no change of tau secretion was noted by neurons treated with 10mM KCl indicating that Golgi fragmentation preceded tau secretion (Fig 2D). A similar observation was noted when neurons were treated with 20 mM KCl although tau secretion had the tendency to be increased (Fig 2D). We also compared the percentage of neurons presenting a fragmented Golgi at 1h and 6 h after application of KCl. After 1h of 10 mM and 20 mM KCl treatment, 51.9% and 67.2% of neurons, respectively, presented a fragmented Golgi compared to 41.2% and 50.2% after 6 h. The lower percentage of neurons with a fragmented Golgi at 6h compared to 1h treatment indicated that the Golgi had begun to reassemble and therefore its fragmentation was transitory upon induction of hyperexcitability. All together, the above observations revealed that Golgi dynamics were correlated to an increase of tau secretion.

### Tau secretion is reduced when Golgi fragmentation is prevented by the inhibition of cdk5

**Fig 3:**
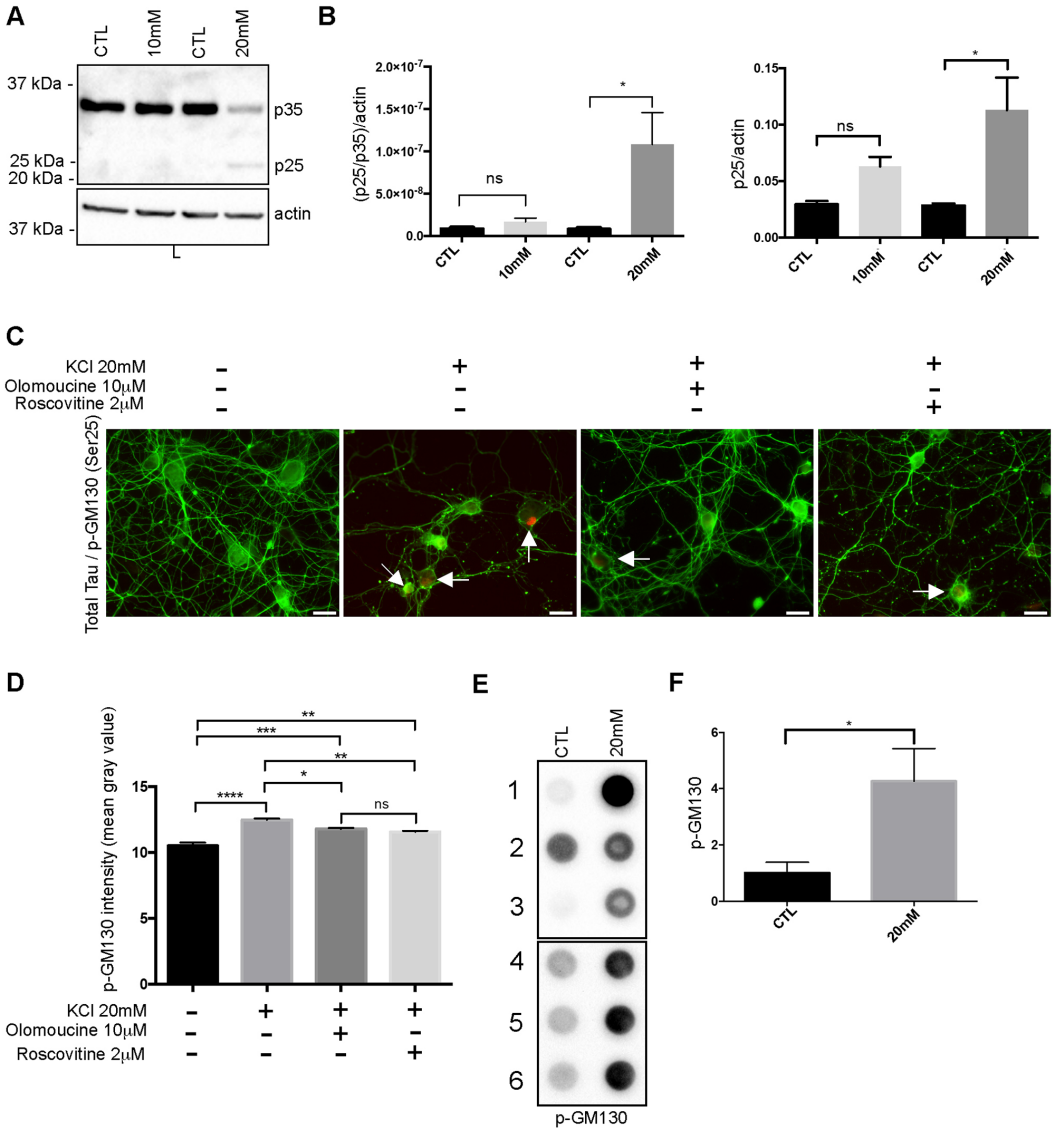
Neuronal hyperexcitability activates cdk5. (A) Immunoblot analysis of cdk5 activity in cell lysate either upon a physiological concentration of KCl (CTL) or elevated concentrations of KCl (10mM or 20mM) by examining the cleavage of p35 to p25. (B) The quantification by densitometry (arbitrary unit) of the ratio p25/p35 revealed that p25 was increased upon hyperexcitability. The ratio was normalized to actin. P25/actin ratio was also calculated. (n = 6, mean ± SEM, one-way ANOVA, Tukey’s test, *P < 0.05). (C) Immunostaining of primary cortical neurons with the anti-phospho-GM130 antibody (red) and the A0024 antibody (green). The phospho-GM130 staining was stronger in neurons treated with an elevated concentration of KCl (arrow) than in control neurons. The phospho-GM130 staining was decreased when neurons were pre-incubated either with olomoucine or roscovitine before treatment with an elevated concentration of KCl. Scale bar = 30μm. Immunostaining of primary cortical neurons with the anti-phospho-GM130 antibody (red) and the A0024 antibody (green). The phospho-GM130 staining was stronger in neurons treated with an elevated concentration of KCl (arrow) than in control neurons. The phospho-GM130 staining was decreased when neurons were pre-incubated either with olomoucine or roscovitine before treatment with an elevated concentration of KCl. Scale bar = 30μm. (D) Quantification of the phospho-GM130 signal intensity using Image J showed an increase of the intensity when cells were treated with a high concentration of KCl. The treatment with olomoucine or roscovitine before KCl treatment decreased the intensity of the signal (n = 3, mean ± SEM, one-way ANOVA, Tukey’s test, *P < 0.05, **P < 0.01, ***P < 0.001, ****P < 0.0001). (E) Dot blot was used to measured the amount of phospho-GM130 in the cell lysate of control neurons and neurons treated with 20 mM KCl. (F) Quantification by densitometry of the phosphor-GM130 signal (n = 6, mean ± SEM, unpaired t-test two-tailed, *P < 0.05).

**Fig 4:**
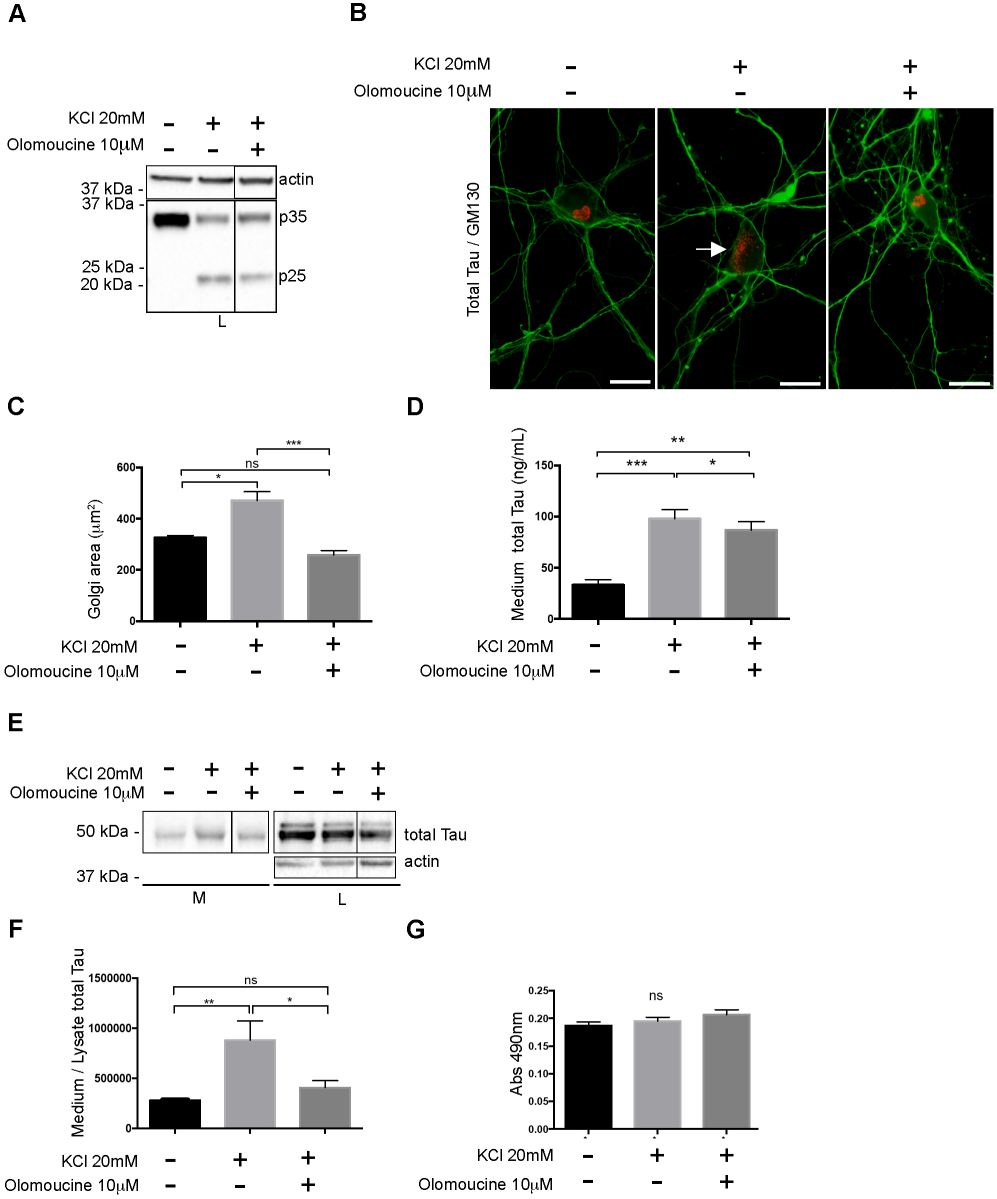
Cdk5 inhibition by olomoucine prevents the fragmentation of the Golgi and decreases Tau secretion. Neurons were pre-incubated for 1h with 10μM olomoucine before being treated with 20mM KCl for 6 h. (A) Immunoblot analysis of p35 cleavage showed that hyperexcitability induction was not affected by the pre-incubation with olomoucine. (B) Immunostaining with the GM130 antibody (red) and the A0024 antibody (green). The Golgi was fragmented when neurons were treated with an elevated concentration of KCl (arrow). The Golgi was not fragmented when neurons were pre-incubated with olomoucine and then treated with an elevated concentration of KCl. Scale bar = 30μm. (C) Quantitative analysis of the surface area of the Golgi complex μm^2^) either upon a physiological concentration of KCl, a high concentration of KCl or a high concentration of KCl + olomoucine. The olomoucine pre-treatment prevented Golgi fragmentation induced by the KCl treatment (n = 5, mean ± SEM, one-way ANOVA, Tukey’s test, *P < 0.05, **P < 0.01, ***P < 0.001). (D) The amount of Tau in the medium was quantified using MSD^®^ MULTI-SPOT mouse total Tau assay. (n = 8, mean ± SEM, one-way ANOVA, Tukey’s test, *P < 0.05, **P < 0.01, ***P < 0.001) (E) Immunoblot analysis of Tau secretion. The increase of Tau secretion induced by a high concentration of KCl was prevented when neurons were pre-treated with olomoucine. (F) Quantification by densitometry (arbitrary unit) of the ratio of total Tau in the medium to total Tau in the cell lysate. (n = 10, mean ± SEM, one-way ANOVA, Tukey’s test, *P < 0.05, **P < 0.01). (G) Cell damage was evaluated by measuring the amount of LDH released in the culture medium. The increase of LDH in the medium was not statistically different (n.s) between all the conditions (n = 10, mean ± SEM, one-way ANOVA, Tukey’s test).

We then examined the signaling pathway leading to the fragmentation of the Golgi upon KCl treatment. Cdk5 activation was shown to result in a fragmentation of the Golgi by Aβ [49]. We therefore examined whether cdk5 was activated in our experimental conditions. Cdk5 is activated at the plasma membrane when it binds to its co-factor p35. In pathological conditions such as the accumulation of Aβ, p35 is cleaved in p25 [50–52]. The binding of cdk5 to p25 results in its constitutive activation and mislocation in the cytoplasm where it can phosphorylate Golgi proteins [51, 52]. Firstly, we examined whether p35 was cleaved in p25. A band corresponding to p25 was only significantly increased in neurons treated with 20mM KCl (Fig 3A and 3B). To further confirm that cdk5 was activated, we examined the phosphorylation state of the Golgi protein GM130, a known substrate of cdk5. Cdk5 phosphorylates GM130 at Serine 25 [53]. Using an antibody directed against GM130 phosphorylated at Serine 25, we noted a stronger staining in neurons treated with high concentrations of KCl than in control neurons (Fig 3C and 3D). This strong phosphorylated GM130 staining was observed in 11.6% and 35.7% of control and KCl treated neurons, respectively. When neurons were pre-treated either with olomoucine or roscovitine, two inhibitors of cdk5, before the KCl treatment, only 15.7% and 13.4% of neurons presented phosphorylated GM130 [54, 55]. The increase of phosphorylated GM130 signal per neuron was also quantified. A significant increase was noted in neurons treated with high concentration of KCl (Fig 3D). However, this increase was significantly reduced in neurons pre-treated with olomoucine or roscovitine (Fig 3D). The increase of GM130 phosphorylation in neurons treated with 20mM KCl was also noted in cell lysates using dot blotting (Fig 3E and 3F). We then examined whether the fragmentation of the Golgi was blocked when neurons were pre-treated with olomoucine before the KCl treatment. This inhibitor prevents cdk5 activation by acting as a competitive inhibitor for ATP [54]. Neurons were pre-incubated with 10μm olomoucine for 1 h before the treatment with 20mM KCl. After an incubation of 6 h with KCl, neurons were fixed to examine whether olomoucine could prevent the fragmentation of the Golgi induced by hyperexcitability. To make sure that the treatment with olomoucine did not affect the induction of hyperexcitability by an elevated concentration of KCl, the cleavage of p35 was examined in neurons pre-treated with olomoucine. As expected, p35 was cleaved confirming that hyperexcitability was induced in neurons treated with both olomoucine and a high concentration of KCl (Fig 4A). As shown in Fig 4B, neurons treated with both olomoucine and 20mM KCl presented a Golgi that was more compacted than neurons only treated with 20mM KCl as revealed by GM130 staining. The Golgi area of neurons treated with olomoucine was similar to that of control neurons (Fig 4C). Lastly, the percentage of neurons presenting a fragmented Golgi decreased with the olomoucine treatment (~20%) compared to neurons only treated with KCl (~51%).

The above experiments demonstrated that olomoucine could be used to block the fragmentation of the Golgi induced by hyperexcitability. Based on this, olomoucine was used to demonstrate that the Golgi fragmentation was linked to the increase of tau secretion induced by hyperpexcitability. Both the amount of Tau detected by the MSD MULTI-SPOT assay and the amount of Tau immunoprecipitated from the culture medium revealed that Tau secretion was decreased when neurons were pre-treated with olomoucine before the induction of hyperexcitability (Fig 4D, 4E and 4F). When neurons were only treated with olomoucine, no decrease of tau secretion and fragmentation of the Golgi were detected (S2A and S2B Fig). LDH was measured in the medium to show that neurons were not damaged by the drug treatment (Fig 4G).

To make sure that the effects on the fragmentation of the Golgi and Tau secretion were not specific to olomoucine, another cdk5 inhibitor, roscovitine, was used (S3 Fig). The Golgi area was decreased by roscovitine pre-treatment (S3A Fig). Consistent with this, the percentage of neurons presenting a fragmented Golgi decreased with the roscovitine treatment (~21%) compared to neurons only treated with KCl (~51%). Finally, Tau secretion was decreased when neurons were pre-treated with roscovitine (S3B and S3C Fig). The amount of LDH in the medium revealed that no cell damage was induced by the roscovitine treatment (S3D Fig). All together, the above observations indicated that the Golgi fragmentation could contribute to the increase of Tau secretion induced by hyperexcitability.

**Fig 5:**
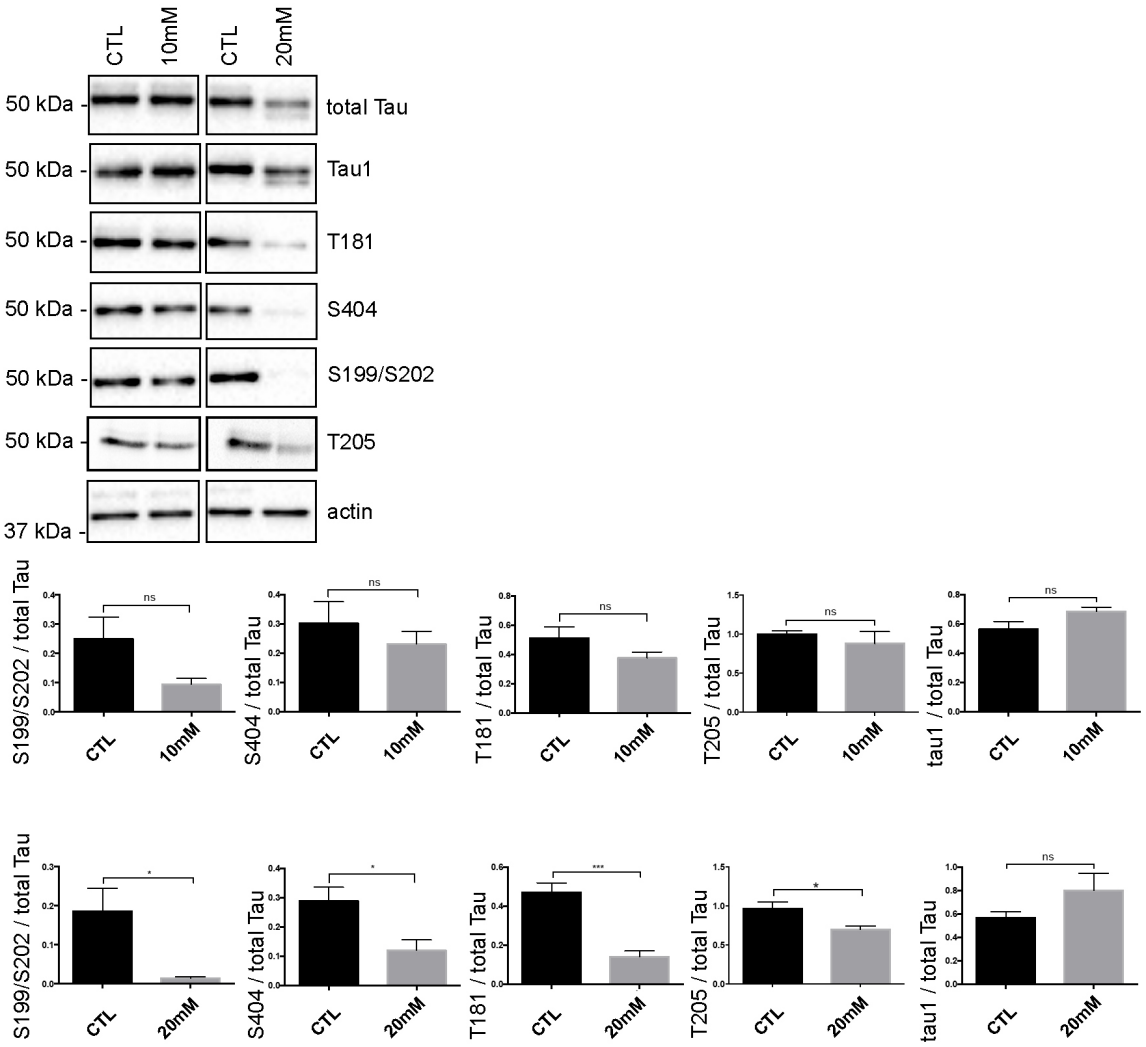
Tau phosphorylation is decreased in neurons treated with 20 mM KCl. Immunoblot analysis of Tau phosphorylation in neurons treated either with 10mM or 20mM KCl. The phosphorylation of S199/S202, T181, T205 and S404 was not significantly affected by 10mM of KCl treatment whereas the phosphorylation of all these sites was decreased by 20mM of KCl treatment. The signal of the phospho-antibodies was normalized to that of total Tau (n = 5, mean ± SEM, unpaired t-test two-tailed, *P < 0.05, ***P < 0.001). The amount of dephosphorylated Tau revealed by the Tau-1 antibody was normalized to that of total Tau lysate (n = 5, mean ± SEM, unpaired t-test two-tailed, *P < 0.05).

Since Cdk5 was activated and that it is known to phosphorylate tau, we examined whether tau phosphorylation was affected by hyperexcitability. In the cell lysate, two main bands at 52 and 57 kDa corresponding to monomeric full-length Tau were detected by the A0024 antibody in control and treated neurons (Fig 5). Tau phosphorylation was not significantly modified when neurons were treated with 10 mM KCl. Surprisingly, Tau phosphorylation was significantly decreased at threonine (T) 181, serine (S)199/S202, T205 and S404 in neurons treated with 20 mM KCl (Fig 5). The staining of the Tau-1 antibody recognizing unphosphorylated tau was not changed when neurons were treated with 10 mM KCl but it had a tendency to be increased in neurons treated with 20 mM KCl (Fig 5). The phosphorylation state of Tau in the medium seemed to be extremely low since no signal was detected with the anti-phospho tau antibodies.

### Suppression of Rab1A induces a Golgi fragmentation and an increase of Tau secretion by neurons and HeLa cells

**Fig 6:**
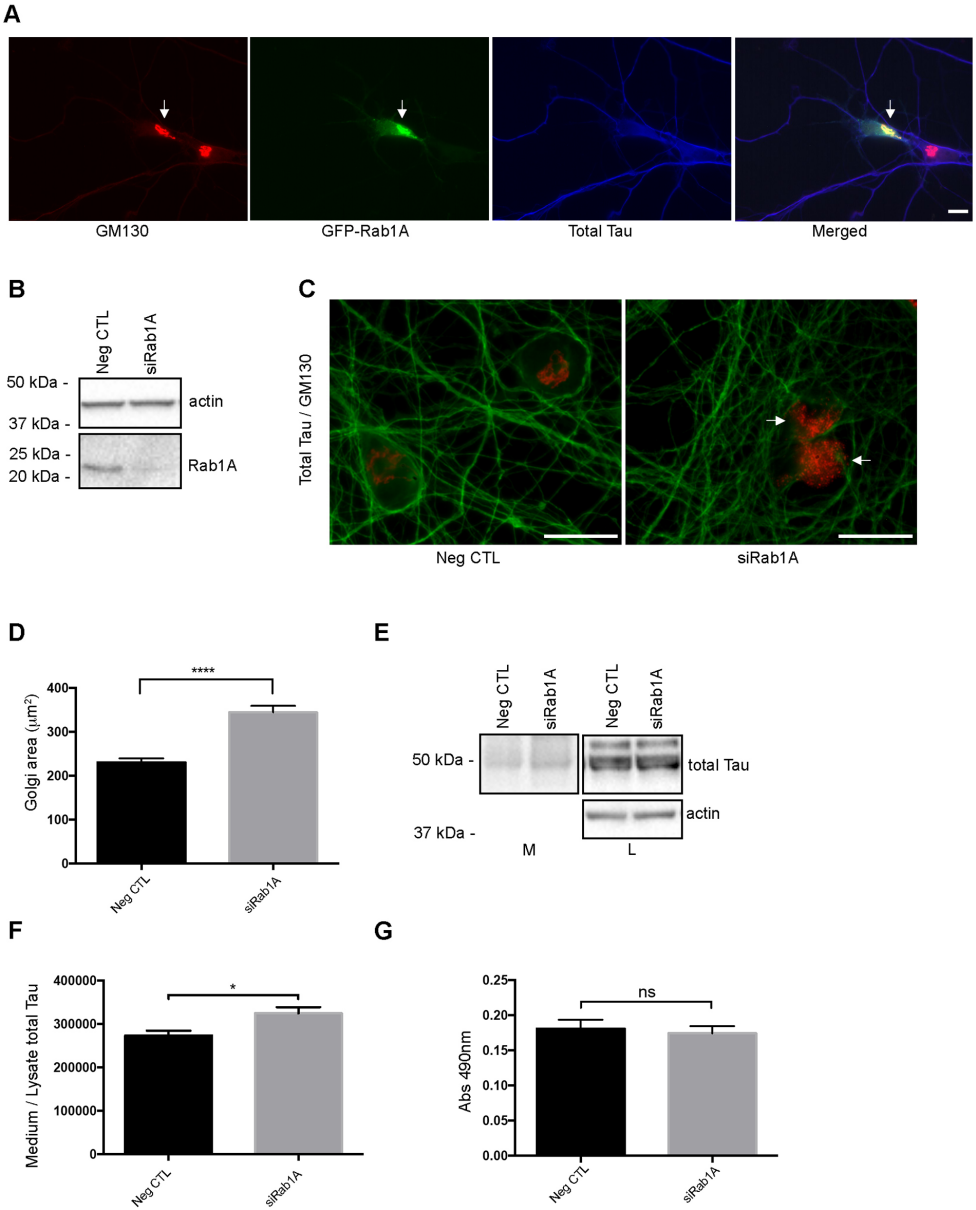
Suppression of Rab1A increases Golgi fragmentation and Tau secretion in primary cortical neurons. (A) GFP-Rab1A co-localizes with GM130 in primary cortical neurons (arrow). Scale bar = 30μm. (B) The knockdown of Rab1A was confirmed by immunoblotting in neurons. (C) Immunostaining of the GM130 antibody (red) and the A0024 antibody (green). The Golgi was fragmented when Rab1A was suppressed in neurons. Scale bar = 30μm. (D) Quantification of the surface area of the Golgi complex (μm^2^) when Rab1A was suppressed in neurons. (n = 7, mean ± SEM, unpaired t-test two tailed, ****P < 0.0001) (E) Immunoblot analysis of Tau secretion in neurons treated either with *Rab1A* siRNA or control siRNA (NegCTL). (F) Tau secretion was increased upon Rab1A suppression in neurons. Quantification by densitometry (arbitrary unit) of the ratio of total Tau in the medium to total Tau in the cell lysate. (n = 5, mean ± SEM, unpaired t-test two-tailed, **P < 0.01). (G) Cell damage was evaluated by measuring the amount of LDH released in the culture medium. The amount of LDH in the medium was not affected by the suppression of Rab1A (ns). (n = 5, mean ± SEM, unpaired t-test two-tailed).

**Fig 7:**
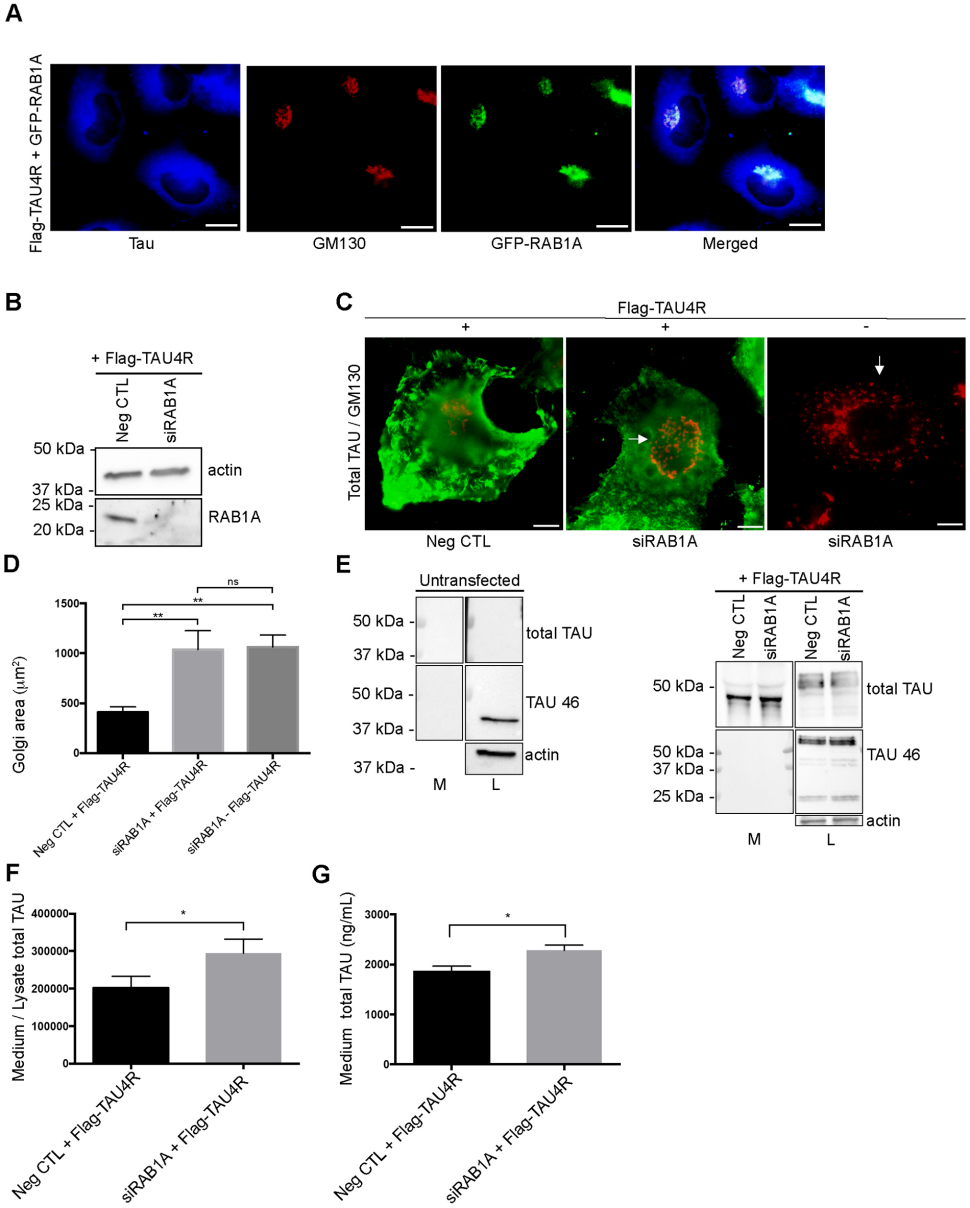
Suppression of RAB1A increases Golgi fragmentation and Tau secretion in HeLa cells. (A) Rab1A co-localizes with GM130 in primary cortical neurons in HeLa cells overexpressing GFP-RAB1A and Flag-TAU4R. Scale bar = 30μm. (B) The knockdown of RAB1A was confirmed by immunoblotting in HeLa cells transfected with wild-type human TAU (Flag-TAU4R) and *RAB1A* siRNA (siRab1A). (C) Immunostaining of the GM130 antibody (red) and the A0024 antibody (green). The Golgi was fragmented when RAB1A was suppressed in HeLa cells either with or without Flag-TAU4R overexpression. Scale bar = 20μm. (D) Quantification of the surface area of the Golgi complex (μm^2^) when RAB1A was suppressed either with or without Flag-TAU4R overexpression. (n = 9, mean ± SEM, one-way ANOVA, Tukey’s test, **P < 0.01). (E) Immunoblot analysis of TAU secretion by HeLa cells transfected with either Flag-TAU4R and *RAB1A* siRNA or Flag-TAU4R and control siRNA (NegCTL). No Tau signal was detected in the cell lysate and medium of untransfected HeLa cells. TAU secreted by HeLa cells was cleaved at the C-terminal as revealed by its absence of immunoreactivity to the Tau46 antibody. A non-specific band just above 37 kDa was noted in the cell lysate of untransfected cells revealed with the Tau46 antibody. In transfected cells, a TAU-positive band was detected at around 50 kDa in the cell lysate and below 50 kDa in the culture medium by the A0024 antibody (total Tau). The Tau46 antibody only detected TAU in the cell lysate. (F) TAU secretion was increased upon RAB1A suppression in HeLa cells. Quantification by densitometry (arbitrary unit) of the ratio of total TAU in the medium to total TAU in the cell lysate. (n = 5, mean ± SEM, paired t-test two-tailed, *P < 0.05). (G) The amount of TAU in the medium was quantified using MSD MULTI-SPOT total Tau assay. (n = 5, mean ± SEM, unpaired t-test two tailed, *P < 0.05).

The above results suggested that Tau secretion was influenced by Golgi dynamics. To confirm this, we verified whether Golgi fragmentation not induced by hyperexcitability could modulate Tau secretion. The Rab proteins, small GTPases involved in membrane trafficking, play pivotal roles in the maintenance of the Golgi morphology [56]. For example, the suppression of Rab1A involved in the ER-to-Golgi trafficking, was shown to induce a fragmentation of the Golgi whereas its overexpression had the opposite effect [41, 57]. Firstly, we examined the distribution of Rab1A in primary cortical neurons by transfecting them with GFP-Rab1A construct (Fig 6A). As expected, Rab1A was enriched at the Golgi and co-localized with GM130. Rab1A was then suppressed in primary cortical neurons to examine whether Tau secretion was affected by directly acting on Golgi dynamics. To do this, neurons were transfected with *Rab1A* siRNA at 4 days after plating and the medium was collected 4 days later to examine the amount of secreted Tau. The knockdown of Rab1A was confirmed by western blotting (Fig 6B). Neurons were immunostained with GM130 to monitor the induction of the Golgi fragmentation upon Rab1A suppression (Fig 6C). The Golgi area was significantly increased indicating that the Golgi was fragmented by Rab1A suppression (Fig 6D). As expected, Tau secretion was increased upon Rab1A suppression (Fig 6E and 6F). No difference of LDH amount in the medium was noted between neurons treated either with control siRNA or *Rab1A* siRNA indicating that the neurons were not damaged by siRNA treatment (Fig 6G). We also verified whether p35 was cleaved in p25 upon the fragmentation of the Golgi by Rab1A suppression as noted with 20 mM KCl treatment. No cleavage was observed indicating that cdk5 was not activated and therefore involved in the Golgi fragmentation induced by Rab1A suppression (S4 Fig). Secondly, we examined whether an increase of the Golgi fragmentation by suppression of RAB1A would increase TAU secretion by HeLa cells. Indeed, we previously reported that human TAU was secreted by HeLa cells [9]. Most importantly, TAU secretion by these cells shares several similarities with that of neurons. Tau released by both neurons and HeLa cells is dephosphorylated compared to intracellular Tau, is mainly membrane-free and is secreted by unconventional secretory pathways [9, 10, 30]. As noted in neurons, Rab1A was enriched at the Golgi in HeLa cells (Fig 7A). The knockdown of RAB1A was confirmed by western blotting (Fig 7B). As expected, the Golgi was fragmented in cells treated with *RAB1A* siRNAs as revealed by both GM130 immunofluorescence and a morphological analysis of the Golgi area (Fig 7C and 7D). Then, we examined whether the induction of the Golgi fragmentation by the suppression of RAB1A had an effect on TAU secretion. To do this, HeLa cells were transfected with *RAB1A* siRNA 24 h before human TAU was overexpressed to make sure that the Golgi was fragmented when TAU expression began. The induction of the Golgi fragmentation by suppression of RAB1A resulted in an increase of TAU secretion (Fig 7E–7G). As noted in our previous study, TAU secreted by HeLa cells was cleaved at the C-terminal as revealed by its absence of immunoreactivity to the antibody Tau46 recognizing an epitope located between the amino acids 428–441 (Plouffe et al) (Fig 7E). The increase of TAU in the medium was also revealed by the MSD MULTI-SPOT total Tau assay (Fig 7G). Collectively, the above observations revealed that by directly acting on the Golgi dynamics through Rab1A, Tau secretion could be modulated.

## Discussion

In the present study, we demonstrated that Golgi dynamics were linked to a modulation of Tau secretion by both primary cortical neurons and HeLa cells. Indeed, the increased Tau secretion induced by neuronal hyperexcitability could be reduced by preventing the Golgi fragmentation through the inactivation of cdk5. The suppression of Rab1A inducing a fragmentation of the Golgi increased Tau secretion by both HeLa cells and neurons. In AD where a Golgi fragmentation is noted, one can speculate that it could correspond to the increased release of TAU by neurons, which would correlate with its accumulation in the CSF and the propagation of its pathology in the brain.

The Golgi apparatus is composed of stacks of flattened discrete membrane-bound compartments called cisternae [37]. However, a fragmentation of the Golgi corresponding to its reorganization into multiple disconnected structures was observed in both physiological and pathological conditions. For example, a fragmentation of the Golgi was reported during migration and division of non-neuronal cells [40, 58]. In neurons, a fragmentation of the Golgi was noted in pathological conditions such as neurodegenerative diseases including AD, Parkinson’s disease and amyotrophic lateral sclerosis [39, 41]. In non-neuronal cells, it is believed that the Golgi fragmentation is a compensatory reaction allowing rapid transport of proteins to their final destination when a cell undergoes a major reorganization to adapt to novel needs. However, it remains unclear whether the fragmentation of the Golgi in neurodegenerative diseases is beneficial or detrimental to neurons. In AD, a fragmentation of the Golgi was reported to perturb the processing of amyloid precursor protein (APP) resulting in an increased production of Aβ, which in turn further fragments the Golgi [49]. Here, we report that the Golgi fragmentation preceded the increase of Tau secretion induced by hyperexcitability indicating that it could contribute to Tau release by neurons. Interestingly, our data also revealed that the Golgi fragmentation induced by hyperexcitability would be transitory indicating that any insult increasing Golgi dynamics could exert an effect on Tau secretion. Since extracellular Tau is linked to the propagation of Tau pathology in the brain, it would be interesting to investigate whether the Tau forms released by Golgi dynamics contributes to this propagation.

In AD, fragmentation of the Golgi was reported more than a decade ago but the mechanisms involved in such an event remain largely unexplored. The Golgi morphology is regulated by 3 classes of proteins: the microtubules and microtubule-associated motor proteins; the structural Golgi proteins; and the proteins of the Golgi transport machinery [41]. Aβ was shown to induce the fragmentation of the Golgi by the activation of cdk5 that phosphorylates GRASP65, a structural Golgi protein involved in the formation of tubular connections between the Golgi stacks [49]. Here we showed that upon prolonged hyperexcitability, the activation of cdk5 contributes to the fragmentation of the Golgi as noted for Aβ-induced fragmentation indicating that cdk5 could be a common target for the induction of this event in AD. However, in neurons treated with 10mM KCl, no activation of cdk5 was detected indicating that a pathway other than that of cdk5 could be involved in the induction of the Golgi fragmentation upon hyperexcitability. Furthermore, the cdk5 inhibitors, olomoucine and roscovitine that we employed are not specific to this kinase but can also inactivate cycling cdks [59]. Cycling cdks and cdk5 are inversely expressed during neuronal differentiation. Indeed, cycling cdks are active in proliferating neuronal precursor cells and downregulated during neuronal differentiation whereas cdk5 is upregulated during neuronal differentiation [60–62]. Based on the above observations, cdk5 seems to be the most activated cdks in post-mitotic neurons and therefore the effects on Tau secretion that were observed upon treatment with olomoucine and roscovitine were most likely caused by its inhibition.

The link between Tau pathology and the fragmentation of the Golgi was also examined in both Tau transgenic mice and AD brain. In a previous study, we reported that in JNPL3 mice overexpressing the mutated form of tau, P301L, the Golgi fragmentation was observed in neurons presenting an accumulation of hyperphosphorylated Tau in the soma [46]. The percentage of neurons with a fragmented Golgi was further increased in older mice presenting aggregated Tau [46]. These observations indicated that the Golgi fragmentation was initiated when Tau became hyperphosphorylated. Interestingly, a recent study reported that in AD, the Golgi fragmentation correlates with the accumulation of hyperphosphorylated TAU [45]. However, the mechanisms involved in the Golgi fragmentation correlated to Tau hyperphosphorylation remain to be elucidated. Since microtubules and motor proteins play important roles in the maintenance of the Golgi structure and knowing that Tau affects the stability of microtubules and the binding of motor proteins to microtubules, one can speculate that Tau dysfunction can indirectly alter the Golgi structure through its effects on the microtubule network [63, 64]. However, a direct role of Tau in the Golgi fragmentation cannot be excluded, since we reported that Tau was found at the surface of the Golgi where it could play a direct role in maintaining its morphology by participating in its association with microtubules [65]. This remains to be demonstrated.

Our data revealed that cdk5 activation induced by hyperexcitability did not result in an increase of Tau phosphorylation as expected since cdk5 is known to phosphorylate Tau [66]. This could be explained by the fact that in our experimental conditions where a fragmentation of the Golgi was induced, the Golgi proteins, GM130 and GRASPs, were better substrates for cdk5 than Tau [49, 53]. This is supported by the fact that there was an increase of GM130 phosphorylation at Serine 25, a site phosphorylated by cdk5 when neurons were treated with an elevated concentration of KCl [53]. Surprisingly, Tau was dephosphorylated when neurons were treated with 20 mM KCl. However, we did not observe a significant increase of the Tau-1 antibody staining recognizing tau dephosphorylated at an epitope that contains S199, S202 and T205 phosphorylation sites [67, 68]. In our experimental conditions, these 3 sites might need to be dephosphorylated to observe an increase of Tau-1 staining. If it is the case, one can speculate that reduced phosphorylation at S199 and S202 when neurons were treated with high concentration of KCl was not sufficient to increase Tau-1 staining. Consistent with this, T205 appeared to be a lot less dephosphorylated than S199/S202 by such a treatment. The protein levels of Tau were also significantly decreased in neurons treated with 20mM KCl. Protein degradation can be induced upon neuronal activity. For example, previous studies reported an increase of protein degradation by depolarization of neurons with KCl in both the pre- and post-synaptic terminals [69, 70]. Upon chronic neuronal activity such as a treatment with 20mM KCl for 6 h, tau might be degraded for allowing axonal remodeling.

We and others reported that Tau secretion mainly involves unconventional secretory pathways [9, 12, 16, 26–28]. It is still unclear whether the secretory pathways of Tau are similar in physiological and pathological conditions. For example, we cannot exclude the possibility that the Golgi contributes to Tau release in normal conditions even if its fragmentation is only observed in neurodegenerative diseases. In any case, the important question that remains to be answered is how the secretion of Tau is increased by the fragmentation of the Golgi. Upon this event, Tau could be secreted by vesicles forming at the Golgi that would fuse with the plasma membrane to release their contents. Our results support this. In both HeLa cells and neurons, Tau secretion was increased when Rab1A was suppressed, a condition inducing increased trafficking from the Golgi to the plasma membrane [71]. Based on this observation and the fact that Tau was found at the surface of the Golgi membranes, it can be speculated that Tau-containing vesicles originating at the Golgi could travel along the axon to deliver Tau at the presynaptic terminal [65]. These vesicles could fuse directly or indirectly through endosomes with the plasma membrane to release Tau in the extracellular space [7]. If vesicles originating at the Golgi are involved in Tau secretion, Tau, being a cytosolic protein, is most likely attached at the surface of these vesicles. In such a case, upon the fusion of these vesicles with the plasma membrane, Tau would be found on the cytoplasmic side of the plasma membrane. However, an unknown flip-flop event taking place at the plasma membrane could result in the cytoplasmic side of the plasma membrane containing Tau ending up on the extracellular side and thereby making possible the release of Tau outside the cell as reported for annexin A2, known to interact with Tau [72]. Another possibility would be that Tau could reach the lumen of vesicles by a mechanism involving chaperones such as Hsc70 [73, 74]. In such a case, when the vesicles would fuse with the plasma membrane, Tau would be released in the extracellular space. Finally, the Golgi might not be directly involved in Tau secretion. Secondary events occurring in parallel to the Golgi fragmentation could activate the unconventional secretory pathways involved in Tau secretion.

Although our data did not show that the Golgi was directly involved in Tau secretion, the strong correlation between the increase of Golgi dynamics and the increase of Tau secretion indicates that the alterations of Golgi noted in AD could be involved in the propagation of Tau pathology linked to its release in the extracellular space. Finally, since extracellular Tau was shown to increase Aβ production and that Aβ was shown to induce Golgi fragmentation, one can speculate that a vicious cycle would take place increasing the fragmentation of Golgi and thereby Tau secretion and Aβ production [20, 49]. If such a vicious cycle takes place in AD, prevention of the Golgi fragmentation could significantly delay the progression of the disease.

## Supporting information

S1 Fig To demonstrate that the effects of 20 mM KCl were not induced by hypertonic solution but rather by neuronal activity, neurons were treated with 20 mM NaCl. No increase of Tau secretion and Golgi fragmentation were induced by 20 mM NaCl.
(PDF)

S2 Fig Olomoucine does not induce an increase of Tau secretion and Golgi fragmentation. (PDF)

S3 Fig Pre-treatment with roscovitine reduced Golgi fragmentation and Tau secretion induced by 20 mM KCl treatment. (PDF)

S4 Fig Suppression of Rab1A in neurons does not induce the cleavage of p35 in p25. (PDF)

S5 Fig Western blots supporting the results reported in the manuscript. (PDF)
