# Supplementary figures and images for: Tau secretion is correlated to an increase of Golgi dynamics
Source: PLoS One. 2017 May 26;12(5):e0178288. doi: 10.1371/journal.pone.0178288 (PMC5446162; doi:10.1371/journal.pone.0178288)

**A**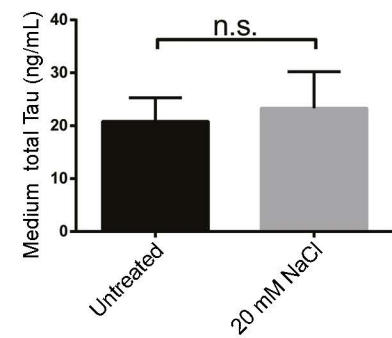**B**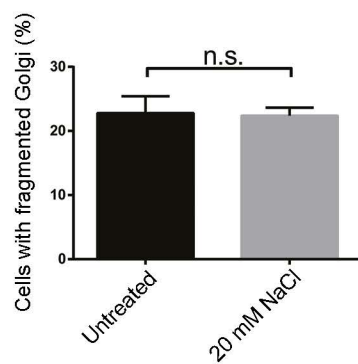

Supplement: S1 Fig — No increase of Tau secretion and Golgi fragmentation were induced by 20 mM NaCl. (PDF) [file pone.0178288.s001.pdf]

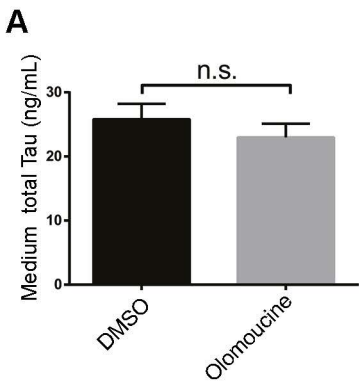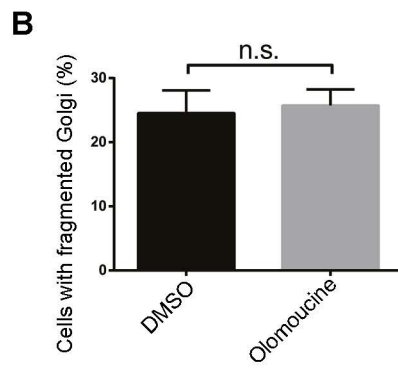

Supplement: S2 Fig — (PDF) [file pone.0178288.s002.pdf]

**A**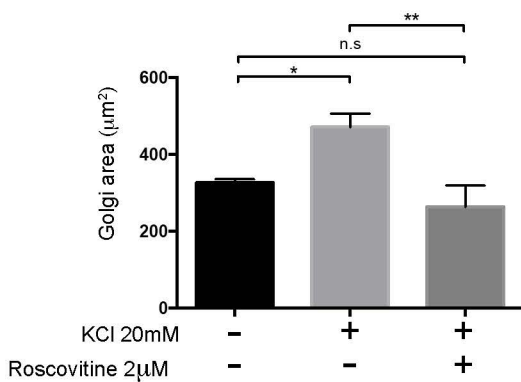**B**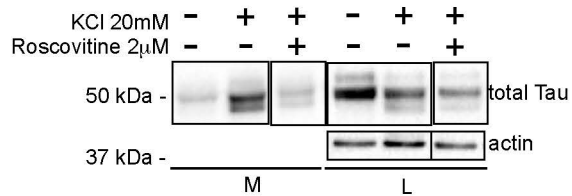**C**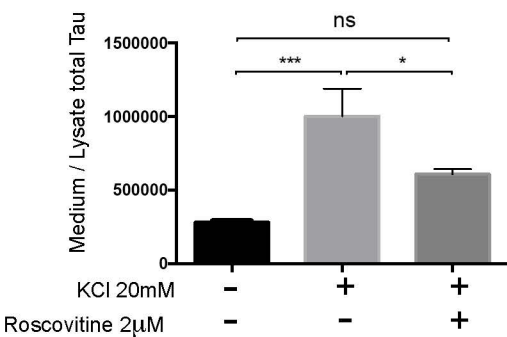**D**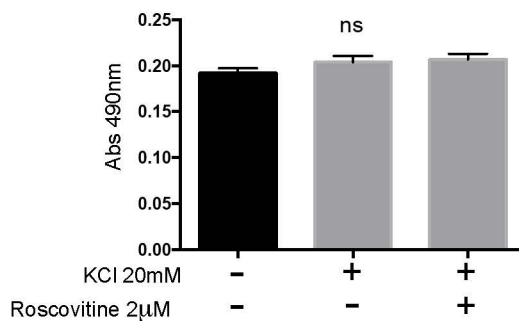

Supplement: S3 Fig — (PDF) [file pone.0178288.s003.pdf]

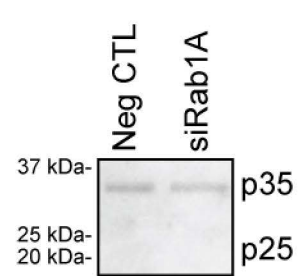

Supplement: S4 Fig — (PDF) [file pone.0178288.s004.pdf]
