# Supplementary material for: Tau secretion is correlated to an increase of Golgi dynamics
Source: PLoS One. 2017 May 26;12(5):e0178288. doi: 10.1371/journal.pone.0178288 (PMC5446162; doi:10.1371/journal.pone.0178288)

Western Blots Fig1A

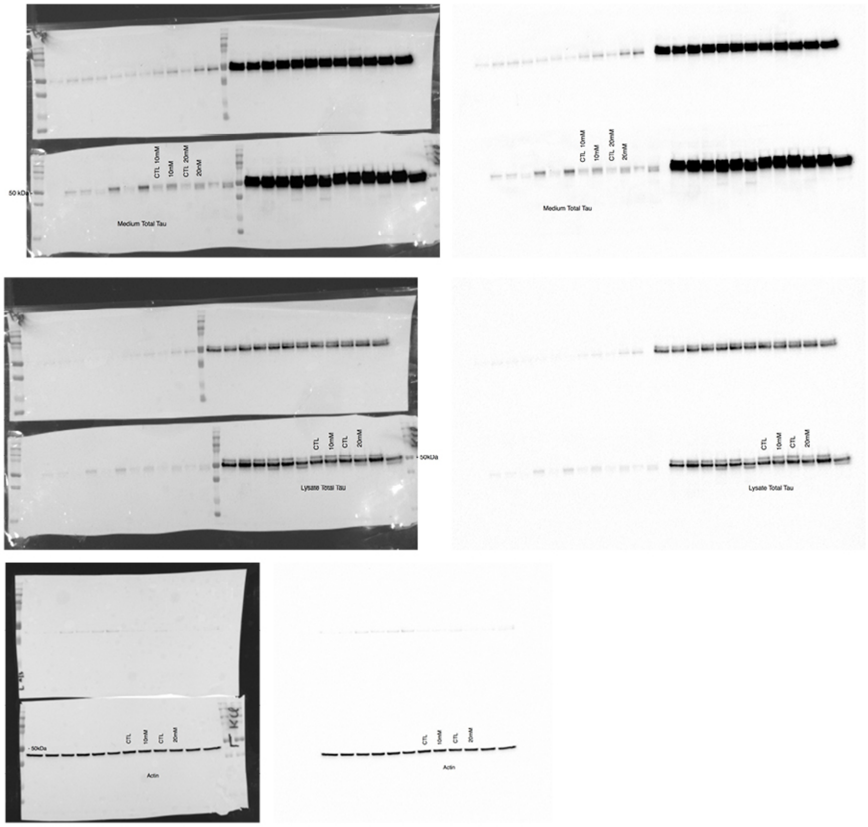

Western Blots Fig3A

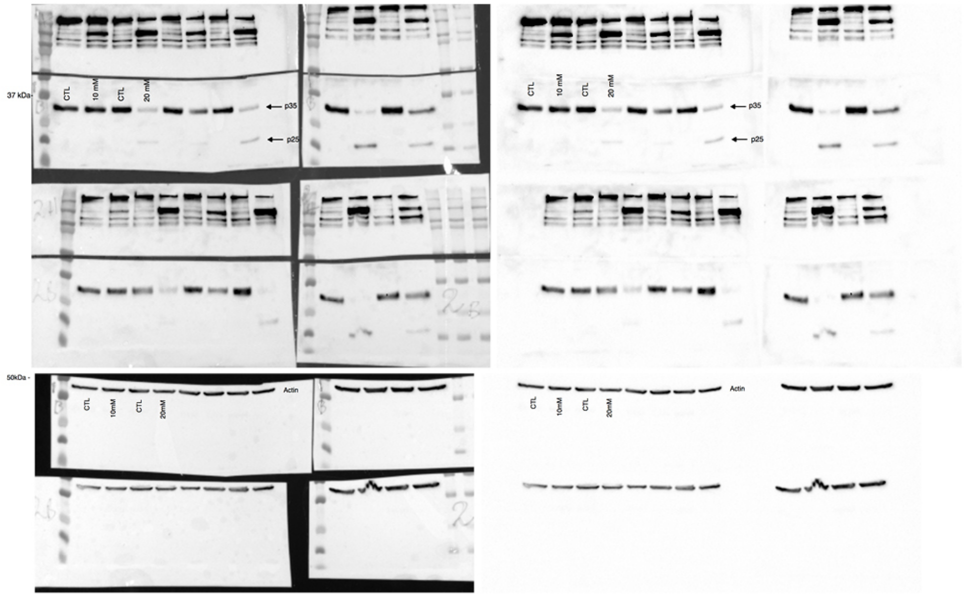

Western Blots Fig4A

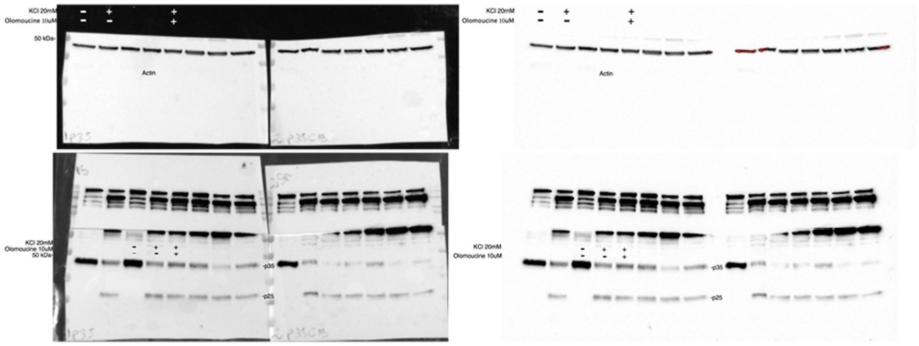

Western Blots Fig4E

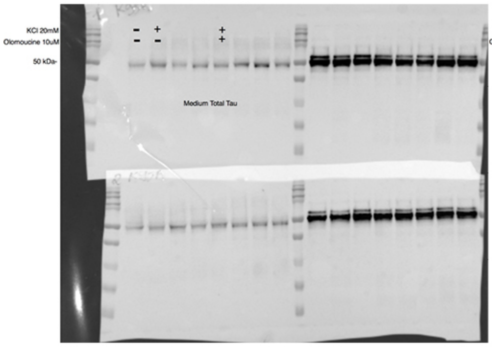

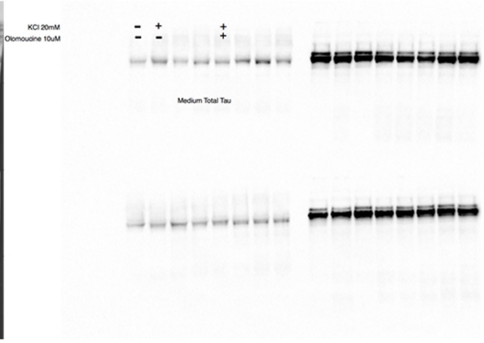

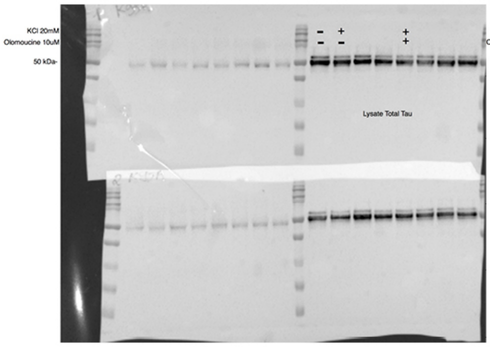

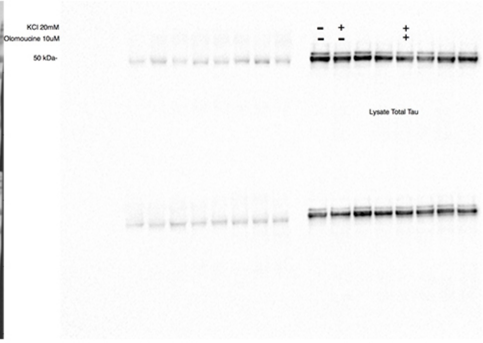

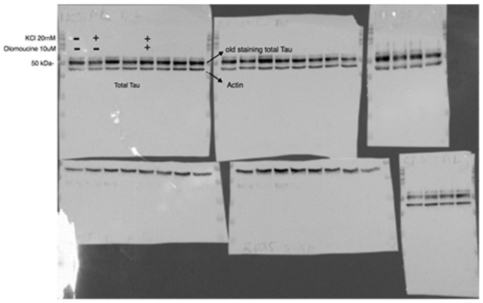

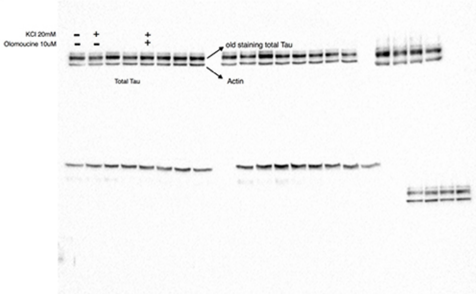

Western Blots Fig5

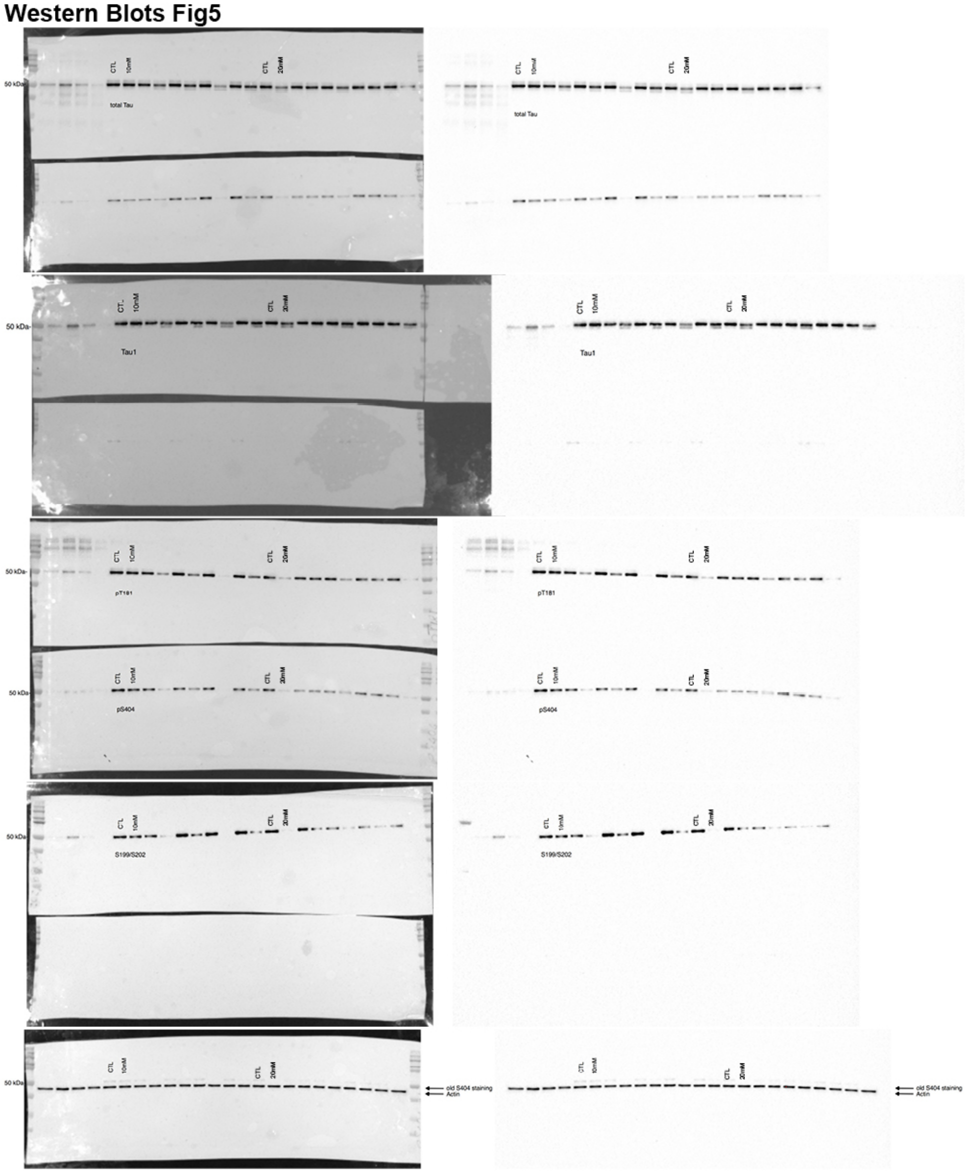

Western Blots Fig5

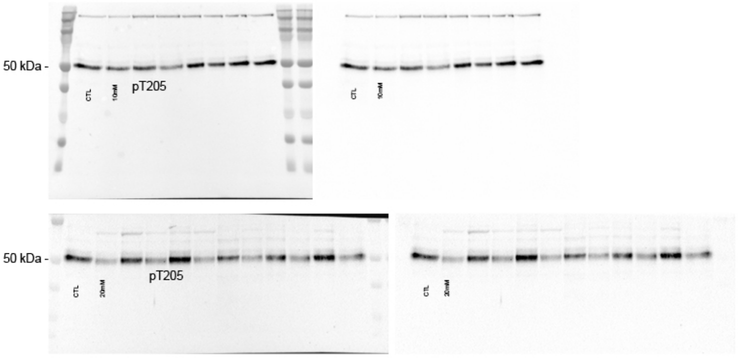

Western Blots Fig6B

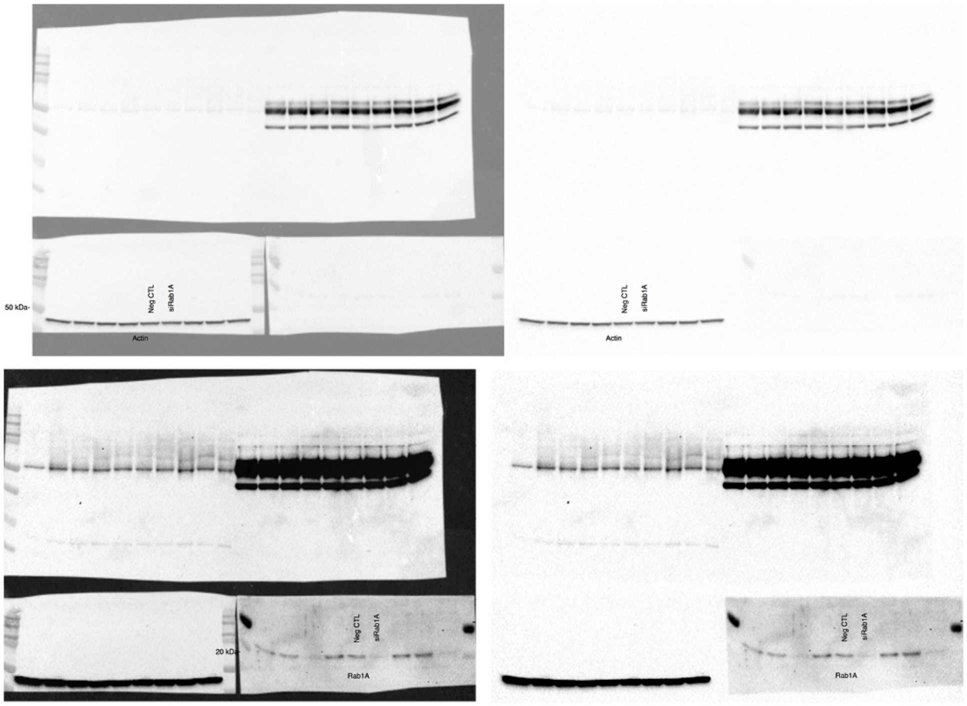

Western Blots Fig6E

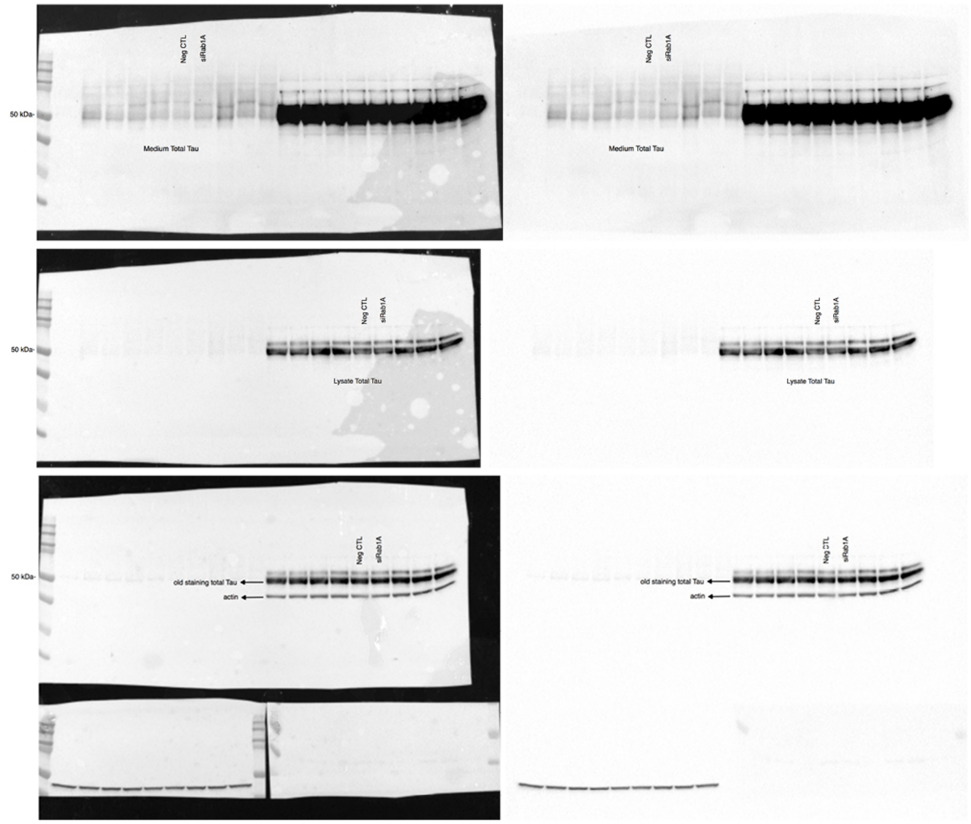

Western Blots Fig7B

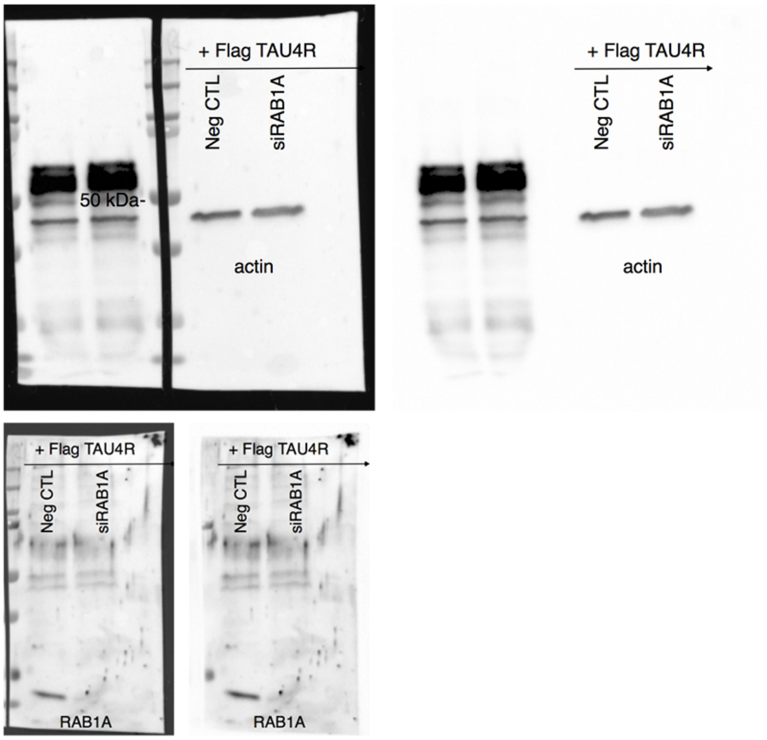

Western Blots Fig7E

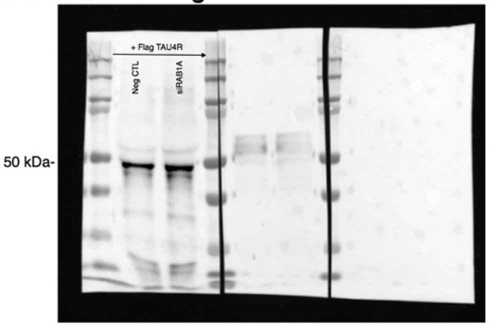

Medium Total TAU

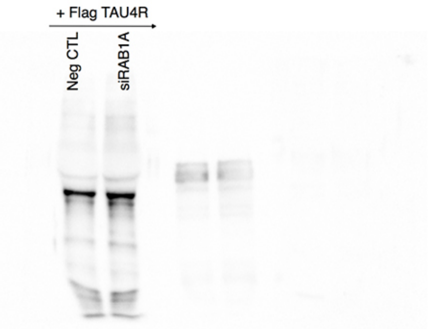

Medium Total TAU

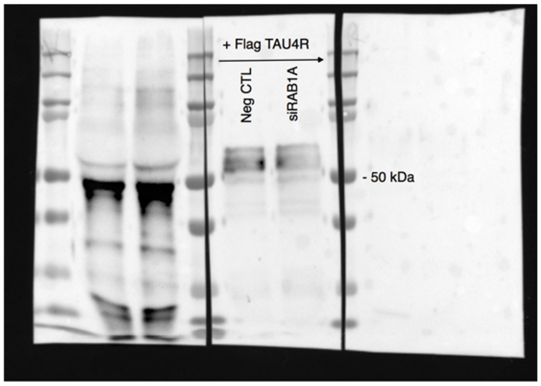

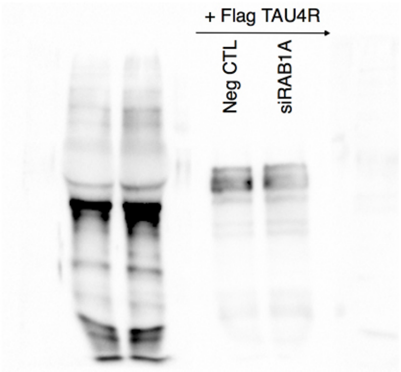

Lysate Total TAU

Lysate Total TAU

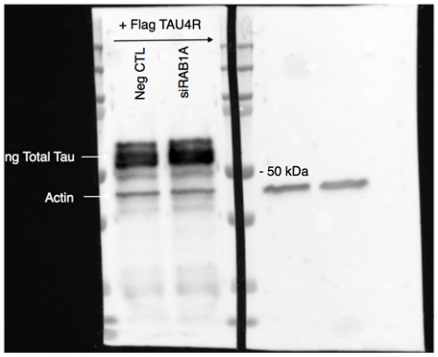

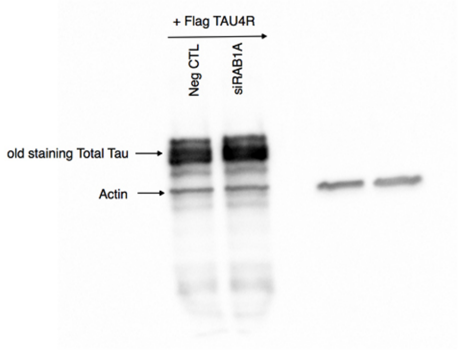

Western Blots Fig7G

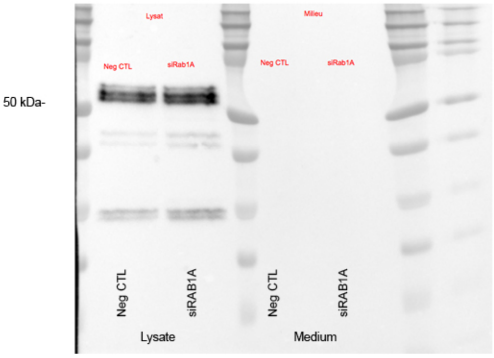

Western Blots FigS2B

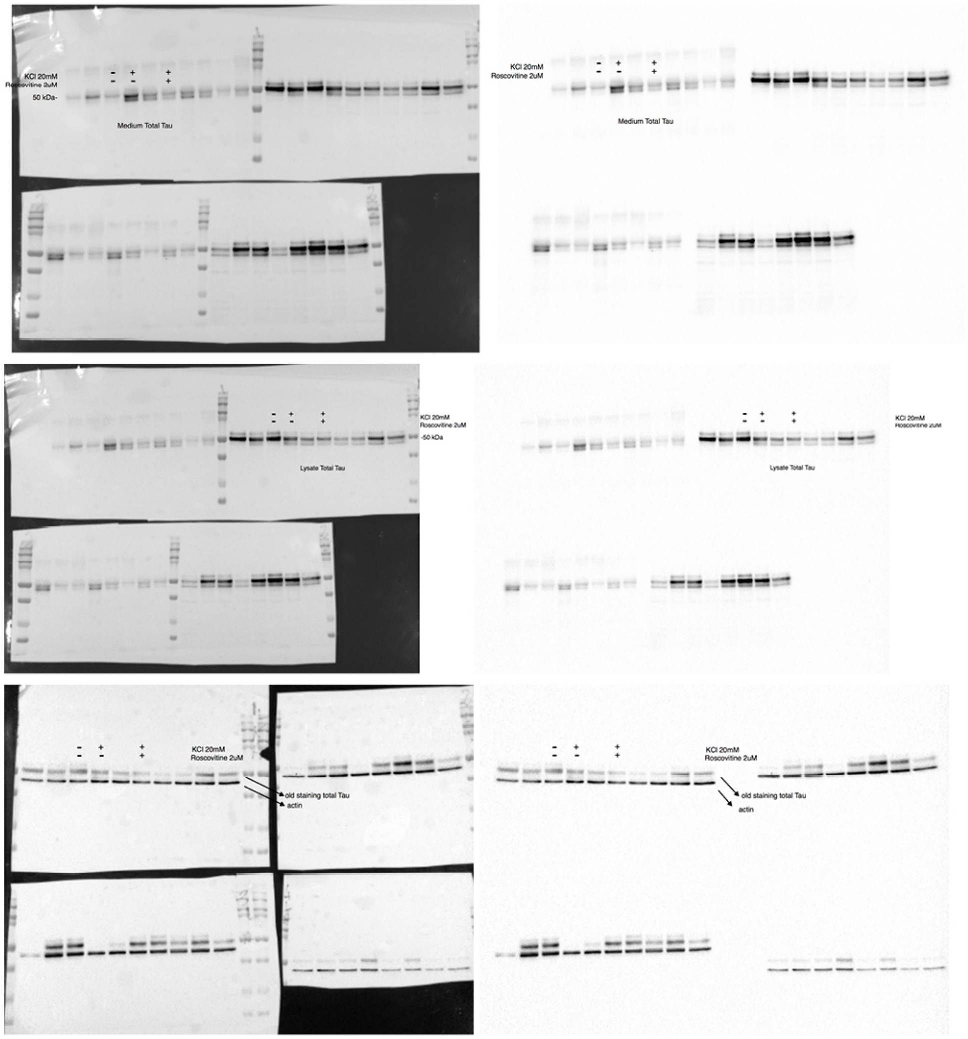

Supplement: S5 Fig — (PDF) [file pone.0178288.s005.pdf]
